# FAIRification of nanosafety data to improve applicability of (Q)SAR approaches: A case study on *in vitro* Comet assay genotoxicity data

**DOI:** 10.1016/j.comtox.2021.100190

**Published:** 2021-11

**Authors:** Cecilia Bossa, Cristina Andreoli, Martine Bakker, Flavia Barone, Isabella De Angelis, Nina Jeliazkova, Penny Nymark, Chiara Laura Battistelli

**Affiliations:** a Environment and Health Department, Istituto Superiore di Sanità, Rome, Italy; b Centre for Safety of Substances and Products, National Institute of Public Health and the Environment (RIVM), Bilthoven, the Netherlands; c Ideaconsult Ltd., Sofia, Bulgaria; d Institute of Environmental Medicine, Karolinska Institutet, Stockholm, Sweden

**Keywords:** AOP, Adverse Outcome Pathway, ECHA, European Chemicals Agency, FAIR, Findable, Accessible, Interoperable, and Reusable, Fpg, Formamido pyrimidine glycosilase, IATA, Integrated Approaches to Testing and Assessment, ISA–Tab, Investigation/Study/Assay Tab-delimited, JRC, Joint Research Centre, Nano-EHS, Nano Environment, Health and Safety, MIRCA, Minimum Information for Reporting Comet Assay, NMBP, Horizon 2020 Advisory Group for Nanotechnologies, Advanced Materials, Biotechnology and Advanced Manufacturing and Processing, NMBP-13-2018 projects, Gov4Nano, NANORIGO and RiskGONE, NMs, nanomaterials, OECD, Organisation for Economic Co-operation and Development, OTM, Olive tail moment, (Q)SAR, (Quantitative) structure-activity relationship, REACH, Registration, Evaluation Authorisation and Restriction of Chemicals, SCGE, Single Cell Gel Electrophoresis, SOPs, Standard Operating Procedures, Nanomaterials, FAIR principles, (Q)SAR approaches, Nanosafety data, Genotoxicity, *in vitro* Comet assay

## Abstract

(Quantitative) structure-activity relationship ([Q]SAR) methodologies are widely applied to predict the (eco)toxicological effects of chemicals, and their use is envisaged in different regulatory frameworks for filling data gaps of untested substances. However, their application to the risk assessment of nanomaterials is still limited, also due to the scarcity of large and curated experimental datasets. Despite a great amount of nanosafety data having been produced over the last decade in international collaborative initiatives, their interpretation, integration and reuse has been hampered by several obstacles, such as poorly described (meta)data, non-standard terminology, lack of harmonized reporting formats and criteria.

Recently, the FAIR (Findable, Accessible, Interoperable, and Reusable) principles have been established to guide the scientific community in good data management and stewardship. The EU H2020 Gov4Nano project, together with other international projects and initiatives, is addressing the challenge of improving nanosafety data FAIRness, for maximizing their availability, understanding, exchange and ultimately their reuse. These efforts are largely supported by the creation of a common Nanosafety Data Interface, which connects a row of project-specific databases applying the eNanoMapper data model. A wide variety of experimental data relating to characterization and effects of nanomaterials are stored in the database; however, the methods, protocols and parameters driving their generation are not fully mature. This article reports the progress of an ongoing case study in the Gov4nano project on the reuse of *in vitro* Comet genotoxicity data, focusing on the issues and challenges encountered in their FAIRification through the eNanoMapper data model. The case study is part of an iterative process in which the FAIRification of data supports the understanding of the phenomena underlying their generation and, ultimately, improves their reusability.

## Highlights

• Nanosafety assessment.
• FAIR data.
• Data Management.
• Genotoxicity and *in vitro* Comet assay.

## Introduction

Nanoscale materials can take on unique properties (*e.g.* optical, magnetic, electrical, etc), which make them particularly suitable for use in a large number of different sectors, such as electronics, agriculture, cosmetics, food and packaging, medicine, textiles [1]. Their beneficial technological impact, which has led to their widespread use, has as a drawback the increasingly urgent need to assess their safety for human health and environment. Unfortunately, the very same properties that make these materials unique and particularly suitable in different fields of application, make their (eco) toxicological evaluation very challenging. In fact, the overall evaluation of nanomaterials (NMs) implies an extensive characterization of a series of physico-chemical parameters, usually not relevant or not applicable in the case of bulk substances, whose variation could have an impact on their reactivity and eventually on their toxicity.

The complexity in the characterization of NMs, unlike conventional chemicals, begins with the definition of chemical identity, which implies, besides chemical composition, the definition of other parameters, such as crystallinity, particle size, particle shape, surface chemistry, specific surface area [2], [3]. Moreover, in the evaluation of their potential toxicological effects through the use of *in vitro* methods, there is the need to consider the dynamics of NMs in media. It is already established that some physicochemical properties of NMs (*e.g.*, surface chemistry, aggregation/agglomeration state, dispersibility) can drastically change in the interaction with the medium, affecting NMs kinetics, bioavailability, and eventually their toxicity [4]. Furthermore, NMs have an inherently ambiguous nature, since their structural properties are characterized by a distribution of values, rather than a single, well-determinable number [5]. These peculiar characteristics add complexity to the challenge of determining the (eco)toxicological effects of NMs.

In this context, with the rapid development of new NMs and their applications, the safety assessment of nanoparticles is lagging behind, along with the knowledge of the nano-specific determinants influencing toxicity and the understanding of the potential hazard for health and environment [6], [7]. This challenging scenario paves the way to the use of Quantitative Structure-Activity Relationship ([Q]SAR) methodologies (*i.e.,* (Q)SAR models, Grouping and read-across approaches as well as other methods based on structure–activity relationships) [8], which could play an increasingly prominent role in elucidating the key drivers of toxicity, in filling toxicological gaps, and in grouping NMs for evaluation or prioritization purposes.

Research on (Q)SAR methodologies applied to NMs has flourished in recent decades, and its advances are reported in many scientific and technical publications and summarized in a series of review articles [5], [9], [10], [11], [12], [13], [14], [15], [16]. The studies confirmed that (Q)SAR approaches applied to NMs (also called nano-QSAR, QNARs, QNTRs, etc…) can be successfully used to predict the physicochemical properties and bioactivity of nanoparticles as well as to assist in the identification of possible mechanisms of toxicity. In addition, the adequacy of applying grouping and read-across approaches for data-gap filling has been proven in a number of publications [13], [17], [18], [19], [20]. On the other hand, despite the intense scientific and regulatory activity leading to the generation of a large amount of experimental data and information on NMs, a scarcity of datasets suitable for making predictions was generally noted, as well as a general lack of structured means to reuse the current wealth of existing nanosafety data [21].

In recent years, significant investments have been made to contribute to the generation of data, information and tools to enable nano- environmental and health assessment [11]. In particular, in the EU a plethora of projects have been financed and are ongoing on these topics [22]. Among them, the Horizon 2020 project Gov4Nano [23] in collaboration with the two other NMBP-13–2018 projects NANORIGO and RiskGONE [24], aim to develop a proof of concept of an efficient and effective risk governance process for nanotechnologies. For this process, reliable data on the safety of nanomaterials for humans and ecosystems are necessary. The improvement of FAIRness (*i.e.*, Findability, Accessibility, Interoperability, and Reusability) of nanosafety data is a specific part of the Gov4Nano project. One of the pillars in this work is the reusability of existing nanosafety data. A harmonized workflow is applied for a number of diverse case-studies to demonstrate the reusability of nanosafety data, progressing from data quality assessment and curation, to translation of data into formats suitable for specific purposes (*e.g.,* for reuse in (Q)SAR based approaches and/or in user-friendly risk assessment tools). These efforts are largely supported by the eNanoMapper data model [25], an open-source chemical substance infrastructure, which include Nano-EHS (Nano Environment, Health and Safety) data, searchable within the overarching Nanosafety Data Interface [26]. The interface includes data generated by past NanoSafety Cluster projects such as NANoREG, NanoTEST, MARINA, ENPRA, NanoGenoTox, caLIBRAte, NanoReg2 [21]. In addition, Gov4Nano initiated the establishment of the GO FAIR AdvancedNano Implementation Network (IN) [27], a network of people actively supporting the implementation of the FAIR principles (Findable, Accessible, Interoperable, Reusable) in the current nanosafety databases (*i.e.*, data on NM physicochemical characteristics, release and exposure, toxicity and functionality).

In this paper, we illustrate a Gov4nano case study on the FAIRification^1^, through the eNanoMapper data model, of *in vitro* Comet test data, for their reuse in (Q)SAR based predictive methods.

The challenges encountered in data FAIRification and those relating to the specific reuse scenario are reported. The growing FAIRness of *in vitro* Comet data hosted in the eNanoMapper database increases the potential of their reuse and supports the understanding of the phenomena that led to their generation.

The reported work can serve as a concrete example of data FAIRification needs, addressing a specific endpoint and reuse scenario.

## FAIR principles

The FAIR principles (Table 1) [28] are a set of principles designed to guide the improvement of the infrastructure supporting the reuse of data. They put specific emphasis on enhancing the ability of machines to automatically find and use the data, and on supporting its reuse by individuals.

**Table 1:** The FAIR Guiding Principles according to Wilkinson *et al*[28].

Findable	Accessible	Interoperable	Reusable
F1. (Meta)data are assigned a globally unique and persistent identifier	A1. (Meta)data are retrievable by their identifier using a standardized communications protocol	I1. (Meta)data use a formal, accessible, shared, and broadly applicable language for knowledge representation	R1. Meta(data) are richly described with a plurality of accurate and relevant attributes
F2. Data are described with rich metadata (defined by R1)	A1.1. The protocol is open, free and universally implementable	I2. (Meta)data use vocabularies that follow FAIR principles	R1.1. (meta)data are released with a clear and accessible data usage license
F3. Metadata clearly and explicitly include the identifier of the data it describes	A1.2. The protocol allows for an authentication and authorization procedure, where necessary	I3. (Meta)data include qualified references to other (meta)data	R1.2. (Meta)data are associated with detailed provenance
F4. (Meta)data are registered or indexed in a searchable resource	A2. Metadata are accessible, even when the data are no longer available		R1.3. (Meta)data meet domain-relevant community standards

The FAIR principles have emerged in response to the realization that research data is not reused to its full potential [29]. The principles support reuse by setting guidelines for four central needs: data needs to be Findable through persistent identifiers and metadata^2^, Accessible through either open or authorized computational means, Interoperable with computational systems and other types of data in order to allow for further analysis and integration (*e.g.*, supporting discovery of complex interrelations, such as structure–activity relationships, or as weight of evidence for decision-making), and finally it needs to be easily Reusable by others, and thus linked to clear licenses for reuse in order to avoid legal issues [28]. In the EU, the FAIR principles have been recognized at policy level and lie at the heart of the European Chemicals strategy and the European Green Deal [30]. FAIR nanosafety data is key, not only to accelerated research, but also in support of safe and sustainable design of nanotechnology and for establishing robust risk governance frameworks for nanomaterials [21], [31]. Digitalized big data is a corner stone for balancing the two sides of the coin in nanotechnology innovation: i) maintaining functionality and reactivity of nanomaterials, while ii) minimizing the risks for hazard to the environment and human beings [32].

In this paper, we investigate the challenges in reusing genotoxicity data in the perspective of the FAIR principles, to highlight the most important needs in the FAIRification process. This exercise builds on the experience of the Horizon 2020 NanoReg2 project [33] on the reuse of available data to implement a grouping strategy to be used within regulatory frameworks [18]. Among genotoxicity tests, the *in vitro* Comet assay is one of the most commonly used for a wide variety of NMs, making it attractive for predictive methods. To this endpoint, the Joint Research Centre (JRC) recently successfully applied the workflow for grouping and read-across proposed in the ECHA REACH guidance which was updated for NMs [13], [17], [34], [35].

### The need for FAIR genotoxicity data

Genotoxicity evaluation is a key element in the safety assessment of chemicals enabling the identification of potential mutagens and/or human carcinogens through the detection of primary DNA lesions and chromosomal damage.

Data on genotoxicity is crucial in risk assessment and bears heavy weight for whether a material is accepted or not for the market. However, even if knowledge of a materials’ potential for genotoxicity is a “no-go” for marketing (*e.g.,* of a consumer product), it is often tested only at the final stages of product innovation when scale-up becomes relevant [36], [37]. Availability of data on genotoxicity at the early stages of innovation has the potential to guide innovators in the choice of material in order to minimize the risk for a “no-go scenario” due to suspected genotoxicity, which, when sufficient evidence from reliable methods is integrated, is a strong indicator of carcinogenicity [38]. Moreover, FAIR genotoxicity data are essential in enabling the implementation and application of (Q)SAR based approaches in supporting risk assessment and assisting in the identification of nano-specific determinants influencing toxicity. Further aspects of efficient reuse of genotoxicity data include the development of Integrated Approaches to Testing and Assessment (IATAs), for example based on Adverse Outcome Pathways (AOPs) [39], [40]. It also provides a strong basis for reduced needs of animal experiments, by supporting early risk assessment in innovation, in line with safe and sustainable by design approaches, especially if coupled to user-friendly risk assessment tools aimed at use within industrial environments [37].

The genotoxic effects induced by nanomaterials can originate from both primary and secondary mechanisms [41 and references therein]. Some NMs can easily cross the cellular membranes [42] and, in some cases, the nuclear membrane, access the chromatin and interact with DNA, therefore causing a primary damage. Moreover, through a secondary mechanism, NMs can exert an oxidative damage activating inflammation processes and/or cellular oxidative stress. The Comet assay is a suitable test to detect the DNA damage caused by exposure to NMs, and the use of an enzyme-modified protocol adds information on the possible mechanism of action.

## The *in vitro* Comet assay

The Comet assay, also called SCGE (Single Cell Gel Electrophoresis), is a rapid and informative method to detect DNA damage at single cell level used on many different cell types. The assay detects single and double strand breaks present in the DNA as a consequence of a direct damage or as intermediate of DNA repair processes and it is successfully applied both in *in vivo* and *in vitro* genotoxicity testing. Moreover, enzyme-modified version of Comet assay can recognize specific DNA alterations such as oxidized bases and turn them into single strand breaks.

In 1988, Singh *et al*. [43] described a simple and highly sensitive technique for quantitation of low levels of DNA damage in single cells after exposure to X-rays and hydrogen peroxide. The origins of the method are rooted in the studies, dating back to the 70 s [44], on the supercoiled structure of DNA described as a looped structure instead of a linear molecule. When both cellular and nuclear membranes of cells are dissolved and histones are removed from DNA by the high salt (NaCl) concentration solution with detergent, the DNA remains supercoiled (as it was around the nucleosome structure) and is called ‘nucleoid’. A single or double strand break relaxes the DNA winding and allows the DNA loops to migrate when subjected to an electrophoretic field [45]. The DNA loop migration causes the nucleoid to resemble the shape of a comet tail [46].

At the beginning of 2000 the expert’s meetings at the International Workshop on Genotoxicity Test Procedures established the first guidelines for *in vitro* and *in vivo* genetic toxicology testing [47], which were then followed by other guidelines for the *in vivo* comet assay, until the publication of OECD guideline TG 489: *In Vivo* Mammalian Alkaline Comet Assay [48]. Exploiting the possibility of evaluating the DNA damage in cells from different organs, this assay has been widely applied in *in vivo* genotoxicity testing.

Although there is no OECD guideline for the *in vitro* Comet assay, fostering the use of non-animal tests in compliance with the 3R principles (Replacement, Reduction, and Refinement) [49], this test has been extensively used to evaluate genotoxicity of chemicals. Moreover, the enzyme-modified assay for oxidative damaged DNA, originally described by Collins *et al*. [50], allows to detect oxidized purine, such as the highly mutagenic lesion 8-oxoguanine, by using Formamido pyrimidine glycosilase (Fpg) a DNA repair bacterial enzyme, as well as oxidized pyrimidines by the addition of endonuclease III. This step increases the sensitivity and specificity of the detected damage because allows to unmask specific silent DNA lesions.

The Comet assay has several advantages: its demonstrated sensitivity to detect low levels of DNA damage; the requirement for small numbers of cells per sample; the possibility to apply it *in vivo* in cells from many different organs; ease of application; the possibility to conduct studies using relatively small amounts of test samples; the relatively short time period (a few days) needed to complete an experiment [51]. Furthermore, the analysis can be automatized, and the protocol is suitable for high throughput techniques. These characteristics are very useful when a large number of chemicals have to be tested. In addition, the *in vitro* Comet assay was recently anchored to a putative AOP for assessment of lung carcinogenicity by nanoparticles together with a number of other assays useful in integrated approaches to testing and assessment [39].

As a matter of fact, in the last years, Comet assay *in vitro* has been widely used for genotoxicity assessment of NMs present on the market. A lot of scientific papers have been published regarding the genotoxicity evaluation of the most widely used NMs and a great amount of *in vitro* Comet assay results is available from recent international collaborative initiatives.

## Methods

### Available data from eNanoMapper

The data available for the case study were retrieved from the Nanoreg2 instance in the Nanosafety Data Interface. They originate from experiments performed in several EU-funded projects (*i.e.*, FP7 NANoREG [52], NanoGenoTox [53], H2020 NanoReg2 [33]). In each project, different partners produced *in vitro* Comet results for different NMs and nanoforms under different experimental conditions. In particular, we focused on experiments selected in NanoReg2 for grouping purposes: *in vitro* Comet experiments performed by various laboratories for different nanoforms of TiO_2_, ZnO and SiO_2_ (a total of 13 nanoforms), in three cell lines (*i.e.,* A549, BEAS-2B, Caco2) and in different conditions (*i.e.,* with and without FPG; with treatment at 3 and 24 h) [54].

The Comet data were searched through the eNanoMapper database, filtering with respect to the nanoforms and the endpoint of interest (*in vitro* Comet assay) and downloading the results in MS Excel format.

### Data reporting templates

The issue of harmonisation of nanosafety data reporting has been addressed by several international projects and initiatives [11]. The ISA-TAB-Nano (Investigation/Study/Assay Tab-delimited extended to Nano) has been proposed as a standardised electronic format to store NMs data and relating them with protocols, Standard Operating Procedures (SOPs) and methods used to their generation [55]. However, because of the low user-friendliness, the use of this format by the investigators was very limited and several project-specific templates were generated and adopted [11], [56]. The EU-funded FP7 project NANoREG has developed, under the JRC’s leadership, a set of Excel templates roughly following the ISA logic, to develop more user-friendly templates for data logging by the experimental scientists [58].

In the present case study, three templates for *in vitro* Comet experiment reporting, produced in recent EU projects, were analysed: the template used in the NanoGenoTox project (called hereafter NanoGenoTox template) [53], [57], the NANoREG data logging template [58] adopted by H2020 Nanoreg2 project (called hereafter NanoReg2 template) and a modified-NANoREG data logging template mostly used by NANoREG partners (called hereafter NANoREG template).

### Comet assay experimental protocol

The following are the general principles of the protocols by which the *in vitro* Comet data were generated.

The *in vitro* Comet assays were performed in three different cell lines (Caco-2, A549, BEAS 2B) following the international validated protocols and literature for standard [47] and enzyme-modified protocol [59] to study DNA strand breaks and oxidized DNA lesions induced by different TiO2, SiO2, and ZnO nanoforms (from JRC Repository [60]). The treatment at 3 and 24 h was performed with 5 different concentrations of NMs (*e.g.,* 0, 1, 10, 50 and 100 µg/ml), following the protocol for NM suspension preparation used in NanoReg project [61]. Chemicals, such as H_2_O_2_, were used for the positive control.

## Results and discussion

Since the establishment of the eNanoMapper data model, numerous EU projects have adopted it (*e.g.*, FP7 NANoREG, FP7 NanoTEST, FP7 MARINA, ENPRA, NanoGenoTox, caLIBRAte, H2020 GRACIOUS, H2020 NanoReg2) [25], [26]. In these projects, an effort to produce collaborative and harmonized experimental results was performed, promoting the use of agreed SOPs for NMs (*e.g.,* SOPs from NanoGenoTox and NANoREG) [62], [63]. In the H2020 NanoReg2 project, these data were to be used to concretely implement the established grouping strategy [18], in order to verify its applicability in regulatory contexts. However, the process of data gathering, curation, integration and translation of the results represented a bottleneck that hindered the success of the grouping exercise. To overcome the obstacles experienced in this and other initiatives, the H2020 Gov4Nano project has undertaken the FAIRification of nanosafety data supported by the eNanoMapper data model. In this paper we describe a case study ongoing in Gov4nano on the reuse of *in vitro* Comet test data for modelling purposes. In what follows, the problems hampering the reuse of existing data are analysed from a FAIR perspective. Similarly to the challenges recently reported by Jeliazkova *et al.* on the reuse of nanosafety data [21], we present below FAIR related issues encountered specifically for *in vitro* Comet assay data (summarized in Table 2). The issues found and the suggested solutions, in many cases addressed through the eNanoMapper data model, can guide the reporting and collection of *in vitro* Comet data and inform the general data FAIRification process. The obstacles encountered in the specific reuse scenario (*i.e.,* reuse in QSAR approaches), mainly related to the interpretation and integration of the experimental results, are highlighted.

**Table 2:** FAIR principles and related issues for *in vitro* Comet assay.

FAIR principle	FAIR related issues	Example /description	Implemented / suggested solutions	Level of resolution in eNanomapper data model*
F1. (Meta)data are assigned a globally unique and persistent identifier	Difficulties in identifying and retrieving information on materials	IDs unambiguously assigned	eNanoMapper implements persistent IDs that allow findability of a great number of studies on the same material	

F2. Data are described with rich metadata (defined by R1 below)	Difficulties in recognizing comparable experiments of the same assay	Non standardized terminology to report metadata	The eNanoMapper implements an open and community-developed semantic vocabulary (*i.e.*, the eNanoMapper ontology)	

F3. Metadata clearly and explicitly include the identifier of the data it describes	(Meta)data not clearly associated to the related experiment	Information on positive /negative controls not available	In eNanoMapper DB, metadata are associated with the relevant dataset through persistent IDs.	

F4. (Meta)data are registered or indexed in a searchable resource	Difficulties in obtaining large datasets for (Q) SAR modelling purposes	Possibility to easily select in a collection of data all those associated with the test of interest	The eNanoMapper semantic model is an indexed searchable data-retrieval system	

A1. (Meta)data are retrievable by their identifier using a standardized communications protocol	Access to data from disperse sources	Experimental results of *in vitro* Comet assay carried out by many different laboratories and collected in projects specific repositories, or in sparse publications	(Meta)data made discoverable through the Nanosafety Data Interface	

A1.2. The protocol allows for an authentication and authorization procedure, where necessary	FAIR data does not mean open data	Protection and promotion of unpublished data	eNanoMapper provides different authorization levels	

I1. (Meta)data use a formal, accessible, shared, and broadly applicable language for knowledge representation.	Need of machine- readable formats	Difficulty in mapping and comparing different data sets	High potential for interoperability through eNanomapper data model [21]	

I2. (Meta)data use vocabularies that follow the FAIR principles	Need of domain specific ontologies	Lack in harmonization of terminology used to report essential parameters related to the experimental results	eNanoMapper implements an open and community-developed semantic vocabulary (*i.e.,* the eNanoMapper ontology)	

I3. (Meta)data include qualified references to other (meta)data	Relevant metadata are often stored separately, making it difficult to retrieve	Information on characterization of NMs in relevant media should be clearly associated with the related study	eNanoMapper data model allows relevant (meta)data to be linked to the experiment record	

R1. Meta(data) are richly described with a plurality of accurate and relevant attributes R1.3. (Meta)data meet domain-relevant community standards	Lack of harmonized reporting formats/ criteria	(Meta)data from different data generators differ qualitatively and quantitatively	Harmonization of templates and guidances based on Minimum Information for Reporting Comet Assay (MIRCA) list [64]	

R1. Meta(data) are richly described with a plurality of accurate and relevant attributes	Difficulties in comparing results with different levels of data processing from different experiments	Results expressed in raw, preprocessed or final-result format	Provide link to appropriate statistical tools	

R1. Meta(data) are richly described with a plurality of accurate and relevant attributes R1.3. (Meta)data meet domain-relevant community standards	Lack of indication of the overall outcome of the Comet experiment (*i.e.*, is there a genotoxic effect?)	How to merge contradictory results from independent experiments?	Domain experts and data generators should provide guidance in the interpretation of the results	

*Level of resolution:  resolved; : not resolved; : work in progress/partially resolved.

### Findability

Findability issues are largely overcome by the use of the Nanosafety Data Interface, where persistent IDs are assigned to the materials from the beginning (Table 2, F1), so that NMs of interest can be easily and univocally identified and related experimental results retrieved. (Meta)data are indexed and searchable (Table 2, F4) and predefined search options are available, in addition to free text search, to support findability of data available in the repository [26]. This functionality relies on key terms relevant for the identification of the different experiments/nanomaterials. However, since the original (meta)data are not uniform in the use of vocabularies, these terms may differ among projects and data providers (Table 2, F2). As an example in the *in vitro* Comet assay data analysed in the case study, different terminology was used to classify the experiments originating from different sources (*i.e.,* different projects or/and data providers). In some experiments imported in the database, the term “COMET” was adopted to describe the “Method” used, whilst in others the same term described the “Protocol” applied. This difference in classification is an example of lack of harmonization in terminology that makes findability of available Comet assays laborious, even in the presence of a searchable repository. Since available Comet *in vitro* data have been generated in the last decade by different laboratories within different research initiatives, and reported in diverse formats and without the use of controlled vocabularies, many inconsistencies in the terminology exist (*e.g.*, naming of materials, endpoint, methods, cell types, concentrations, time points, etc…) [65]. Examples of lack of controlled vocabularies and standardization in the reported data are displayed in Table 2. In order to overcome this type of issue, deep efforts in data cleaning and curation were necessary to correctly annotate the data according to the eNanoMapper domain-specific ontology [21], [25], [66].

Problems related to lack of standardization and misinterpretation of the information to be reported can be overcome upstream by using guided data entry procedures. In the Nanosafety Data platform, harmonized template wizard is being implemented through a user-friendly interface, to facilitate the data import workflow, minimizing user errors [65]. Through a dedicated web form [67], the user can enter (meta)data of the experiment and download the corresponding Excel template, where to finalize the data entry. The Template Wizard incorporates the domain experts’ needs and maps automatically the eNanoMapper data model, meeting the needs of laboratory practices as much as possible. Format constrains and relevant picklists based on eNanoMapper ontology are under development in the Template Wizard [65] specific for the *in vitro* Comet assay (Table 3).

**Table 3:** Issues related to the lack of controlled vocabulary / harmonization in terminology or field formats of the Comet assay data. Most of them can be resolved with the implementation of field format constrains and picklists based on domain-specific ontologies.

Fields name	Findability related issue	Reccomended solution		Examples
		**Picklist/autocomplete**	**Constrain num/textual**	
Nanomaterial type/nanomaterial	Lack of controlled vocabulary	yes		SiO2 Silica Silicon dioxide
Endpoint	Lack of controlled vocabulary	yes		(*in vitro*) Genotoxicity Genotox DNA strand breaks % DNA in tail OTM NET FPG SITES FPG INCUBATION Not reported
Assay/Assay name	Lack of controlled vocabulary	yes		COMET Comet assay OxiSelect Comet assay Comet assay in BEAS 2B cells Comet FPG
Protocol	Lack of controlled vocabulary	yes		COMET COMET-SB COMET- Net Fpg DNA Strand breaks
Cell type/Cell line	Lack of controlled vocabulary	yes		BEAS 2B BEAS-2B BEAS2B
Cell pass/Cell passage	Field format		Numerical	30 p49 P32 + 9
Seeding	Field format		Numerical	10,000 2x10^5^ Not reported
Results (units) mean or median (SD)	Lack of controlled vocabulary /field format	yes (units)	Numerical (results, SD)	% of dna in tail No fpg % Tail DNA No fpg OTM
Treatment	Lack of controlled vocabulary	yes		In vitro stimulation Pos/neg control sample none
Exposure time /incubation time and units	Lack of controlled vocabulary/ field format	yes (units)	Numerical (exposure)	3 h, 24 h Hours, mins
Concentration and unit	Lack of controlled vocabulary / field format	yes (units)	Numerical (concentration)	ug/cm2 or µg/cm^2^ (mg mL^−1^) µg/ml or µg/mL
Medium	Lack of controlled vocabulary	yes	Textual	DMEM, Dulbecco's modified Eagle medium

Another important issue concerns findability of relevant related metadata (Table 2, F3). Although the data available for the case study originate from large international collaborative initiatives with a great effort to produce harmonized experimental results, reporting was influenced to some extent by the practices and needs of individual researchers. Notably, in many cases it was difficult to trace back to negative and positive controls results, which are essential elements of dose–response assessment, without specific advice from data generators. The poor findability of these critical metadata practically reduces the number of experiments potentially reusable for modelling, compared to the number of studies nominally available in the database. Ambiguity in reporting essential information, which can lead to misinterpretations of results, may be avoided by means of data entry procedures such as the Template Wizard cited above.

### Accessibility

Data on *in vitro* Comet assays performed in recent large collaborative initiatives, as well as in most of the current H2020 Nano-EHS projects, are collected and made easily accessible through the Nanosafety data interface (Table 2, A1) [26]. As FAIR data do not imply openly available data by default, both published and unpublished data are hosted by eNanoMapper with different levels of user rights (Table 2, A1.2) [21], [65].

Accessibility is a fundamental prerequisite to identify existing data, whether openly available or not, and hence support accurate data reuse. The large amount of Comet *in vitro* NMs assays recently performed during EU funded projects [26], and hosted in the eNanoMapper database, would otherwise have been hardly accessible for reuse. Experiments carried out by many different laboratories and collected in projects specific repositories, or in sparse publications, are difficult to identify and laborious to collect in a homogeneous dataset [16]. Access to all potentially relevant information from a single source, whether it be physical hosting or linking multiple resources, in order not to miss important clues on the determinants of toxicity, is a crucial aspect for the development of reliable and widely applicable models and predictions.

### Interoperability

Interoperability between different experimental studies carried out on the same endpoint, in the same experimental conditions, on set of diverse NMs, is an essential pre-requisite to obtain suitable datasets for (Q)SAR modelling purposes [9], [68], [69]. A good data model (*i.e.*, “a well-defined framework to describe and structure (meta)data” [27]) is one of the essential requirements to ensure interoperability between experimental data from different sources. eNanoMapper is based on a data model that ensures high potential for interoperability through data serialization into different formats [21], [65] (Table 2, I1). Furthermore, a FAIRification workflow has been developed for the eNanoMapper database to enable the mapping of non-FAIR data (*e.g.*, Excel files) onto the eNanoMapper data model, through ontology lookups on several layers [65].

The need to easily compare experiments from different sources also implies that (meta)data are described by means of harmonized terminology (Table 2, I2). As an example for the *in vitro* Comet assay specific case, the comparison of the ‘Results’ section of NanoReg2 and NanoGenoTox templates (see Methods), is reported in Table 4. Lack in harmonization of terminology adopted to report essential parameters and differences in the information to be reported, seriously hampers interoperability and requires domain-expert advice. In the Nanosafety Data Interface this obstacle is addressed by the implementation of a nanosafety community-developed ontology [66]. The alignment between the experimental data and the semantic model of eNanoMapper is a constantly evolving process.

**Table 4:** Mapping the fields of the ‘Results’ section among NanoReg2 and NanoGenoTox templates.

NanoReg2 template	NanoGenoTox template
Exposure time (h)	Incubation time (h)
NM concentration (µg/ml)	Dose (µg/ml; µg/cm^2^)
% of dna in tail	
As mean or median	
	Corresponding% viability
	No. cells scored
Lysis only - strand breaks (%tail)	No FPG - % Tail DNA
	OTM
Buffer incubation (%tail)	
Fpg incubation (%tail)	FPG - % Tail DNA
Net fpg sites (%tail)	
References to SOPs	Protocol application
Any deviations from standard protocol	Notes
	Treatment

For the interpretation of the toxicological experiments of NMs in general, as well as for the *in vitro* Comet assays, monitoring of critical physical–chemical parameters during the experiment is crucial. For instance, knowledge of dispersion state [70], [61] is an important factor in determining the specific entity interacting with the biological environment [4]. Furthermore, cells healthiness is an essential requisite of *in vitro* experiments and associated cytotoxicity data should be available for control to be able to evaluate the outcome of a genotoxicity assay [71], [72]. These aspects are necessary to keep in mind when building a meaningful (Q)SAR model, to ensure that the outcome to be modelled is actually the endpoint of interest and not the result of confounding factors. The eNanoMapper data model allows relevant (meta)data to be linked to the experiment record, provided that they have been correctly imported in the database. However, this information is often not reported in the original studies or, when available, stored as separate metadata and difficult to retrieve consistently with the Comet experiment (*e.g.*, in projects specific data repositories). This aspect limits the interoperability of data and negatively affects their reusability (Table 2, I3).

### Reusability

Reusability of *in vitro* Comet data for predictive modelling purposes has been hampered by a number of challenges.

The possibility of comparing the experimental conditions used in different experiments is a fundamental prerequisite for their reuse in order to create homogeneous data sets on the same endpoint (Table 2, R1).

For this purpose, we analysed three templates for reporting *in vitro* Comet experiments used to store data in recent EU projects: the NanoGenoTox template [53], the NANoREG template [58] and the NanoReg2 template (see Methods Section).

**Fig. 1:**
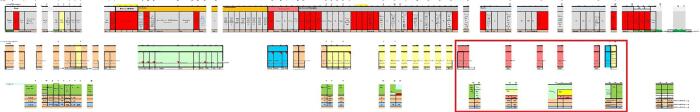
Results of the mapping between templates. Top: NANoREG template [58]; middle: NanoReg2 template; bottom: NanoGenoTox template [53]. The box highlights the ‘Results’ section, reported in detail in Table 4.

The results of the comparison among these templates, consisting of predefined Excel sheets that had to be filled by the data providers, is graphically represented in Fig. 1. In the figure, the correspondent fields of each template are aligned vertically. Blancs represent lack of correspondence among the templates. At a first glance it is evident that the way the experiments are reported differs greatly, both in the number and in the type of fields reported. This is also reflected in Table 5, where the number of fields of each template and the number of common fields among the templates, are reported. For instance, while the template modified by NANoREG partners (NANoREG template) includes a huge number of parameters to be reported (90 fields in total), there is very little overlap with the other templates (having fewer fields: 46 and 28 fields for NanoReg2 template and NanoGenoTox template, respectively. Table 5). These differences strongly limit the comparability of the experimental data, eventually hampering their reusability.

**Table 5:** Number of fields in common between the NANoREG, NanoReg2 and NanoGenoTox templates.

No. common fields	NANoREG template	NanoReg2 template	NanoGenoTox template
NANoREG template	90	39	17
NanoReg2 template	39	46	11
NanoGenoTox template	17	11	28

Challenges of improving reporting formats and criteria for NMs *in vitro* Comet data are manifold. On the one hand, in common with other assays for NMs, the properties that characterize the identity of the material under study and its physical–chemical parameters, both as pristine and in the relevant media, have to be reported. On the other, many differences in reporting criteria stem from the lack of agreement in the scientific community on SOPs for this assay, even for conventional chemicals. As a matter of fact, no OECD guidance it is available for the *in vitro* Comet assay [51], [74], [73] (Table 2, R1.3).

The protocol of Comet assay presents some critical steps for the experimental variability that have been reported in dedicated papers, focusing on interlaboratory differences in baseline and induced DNA damage in the same experimental systems. These critical steps are mainly attributed to the percentage of low melting point agarose, electrophoresis conditions (strength of electrophoretic field and duration of electrophoresis) and scoring method, whereas the duration of lysis treatment is considered less important for the variability of results so that the OECD *in vivo* Comet assay guideline states that the lysis time should be at least 1 h or overnight [51]. The image analysis’ systems, normally used for quantification of DNA damage, provide various types of descriptors, such as “Tail moment”, “Tail length”, “Olive tail moment (OTM)” and “DNA percentage in the Comet tail”. The latter has recently been reported as the easiest to understand and the most meaningful to researchers, usually not familiar with the Comet assay, making it the most used parameter nowadays [75]. As reported in a recent paper [64], these steps of the protocol, identified as critical for determining the level of DNA migration, are often inadequately described in published research articles giving rise to sources of variability. In order to be able to compare the results obtained with the Comet assay in a more effective and reliable way, the hComet Cost Action group of experts [76] have drawn up the Minimum Information for Reporting Comet Assay (MIRCA) recommendations to highlight key aspects of the Comet assay protocol that must be described when reporting the results [64].

Although the MIRCA list is not explicitly designed for NMs testing, it can be used as a starting point for creating harmonized templates and for assessing the completeness of existing ones. Efforts to implement these recent community-defined standards in the *in vitro* Comet reporting templates to be used by data providers, are ongoing in the Gov4nano and NMBP-13–2018 projects.

Another extremely valuable aspect, allowing data reusability in different reuse scenario, is the possibility to access the details, in terms of dose/response results, of each experiment (Table 2, R1). For instance, in regulatory contexts, go back to the original experimental data is often crucial to be able to make a conclusion on the genotoxic effect. Criteria for determining if a genotoxic effect is present or absent generally rely on the detection of i) a statistically significant increase in one dose; ii) a dose-related increase [38], while it should be emphasized that the rules that guide the overall assessment may differ in different contexts (Table 6). Specific guidance and links to statistical tools, to estimate these criteria directly from the original dose–response data, are recommended to improve their reusability.

**Table 6:** Comet assay: examples of rules for the interpretation of results.

	Criteria for assign a genotoxic effect (overall = positive)
OECD TG 489 *in vivo* Comet assay [48] All criteria to be met	i) At least one of the test doses exhibits a statistically significant increase compared with the concurrent negative control;
	ii) the increase is dose-related when evaluated with an appropriate trend test;
	iii) considerations on distribution of historical negative controls.

NanoGenoTox, , *in vitro* Comet assay [62] One of the criteria to be met	i) A statistically significant increase with ≥ 2 doses;
	ii) a statistically significant increase at high dose and a dose-dependent increase.

NANoREG, *in vitro* Comet assay [77] Both criteria to be met	i) A concentration-related induced DNA damage or at least genotoxic response in one concentration with cell viability more than 60% compared to control;
	ii) reproducible response.

NanoReg2, *in vitro* Comet assay [56] One of the criteria to be met (an intermediate category for equivocal results is available)	i) Dose response observed and statistically significant increase at 1 dose (cytotoxicity ≤ 20%);
	ii) statistically significant increase with ≥ 2 doses (cytotoxicity ≤ 20%).

The need for a yes-or-no statement (*i.e.,* presence / absence of genotoxic outcome) is a major issue for the modelers, who often have to manage an (ideally) large number of complex data, without having the appropriate expertise to go into the technical details of the experiments. Furthermore, difficulties in interpretation arise when contradictory results for the same assay (same NM, same experimental conditions) are available, or in presence of contradictory results for the same assay (on the same NM) under different experimental conditions (*e.g.,* different time of exposure, or cell lines used, or enzyme-modified assay).

In the JRC’s grouping and read-across experiment [13], [34], [35], contradictory results were resolved by defining the overall genotoxicity call for each material by the majority call with respect to the *in vitro* Comet assays, that is a genotoxic effect (overall = positive) was assigned when the majority of test results were positive, and an absence of genotoxic effect (overall = negative) when the majority were negative [35]. This strategy, fully acceptable in that context, is not always advisable, as whenever possible conflicting results should be resolved in order to consider the result as not equivocal [78].

Moreover, in different frameworks, such as Safe by Design approaches in pre-regulatory settings, a yes-or-no evaluation on the genotoxicity concern posed by the material is often requested.

Therefore, the reporting, for instance in the eNanoMapper database, of different levels of detail and processing of genotoxicity tests results on the one hand is recommended (Table 2, R1), to cope with different reuse scenarios, on the other hand, in the absence of domain-relevant community standards (Table 2, R1.3), should be thoroughly documented to allow for data interpretation.

## Conclusions

In this paper, the genotoxicity case study served as an example to illustrate the needs and benefits of FAIRification of nanosafety data.

FAIRness of a large variety of genotoxicity data for NMs is increasingly guaranteed by the use of a FAIR infrastructure, such as the eNanoMapper data model, where the problems encountered in managing and harmonizing test data from different sources have been solved with the implementation of community-developed standards (*i.e.,* a domain-specific ontology) and mapping procedures [21]. Moreover, guided data entry procedures are being developed to further minimize possible inconsistencies in reporting of data to be stored in eNanoMapper-supported databases. Despite these significant advances, the reusability of existing genotoxicity data for (Q)SAR-based approaches is still limited in some respects by findability and interoperability challenges. Relevant metadata, which can play an essential role in interpreting the results and resolving contradictory outcomes, are often poorly reported in the original studies. Consequently, their implementation in the database cannot be fully FAIR compliant.

Furthermore, difficulties in obtaining suitable datasets of Comet *in vitro* test data for (Q)SAR based approaches derive from lack of agreement on the minimum set of parameters to be reported to univocally characterize an experiment and ensure its reproducibility and comparability with other similar experiments. In this context, the recently established MIRCA list will serve as a basis to refine and harmonise existing templates [64].

The analysis of existing FAIRified experimental results can help shed light on nano-specific critical parameters whose variation can affect the result of Comet *in vitro* experiments, thus accelerating the standardization of methods and protocols. In an iterative process, increasingly FAIR data support the understanding of phenomena which in turn increases the reusability of the data.

In conclusion, great progress has recently been made towards *in vitro* Comet data reusability, thanks to numerous collaborative initiatives and the development of a FAIR repository to host nanosafety data. However, the process is constantly evolving as is the knowledge of the nano-specific determinants influencing toxicity and the understanding of the hazard posed by nanomaterials to health and environment. To accelerate the FAIRification process, the figure of the data steward prove to be valuable [27]. Such a person’s role as ‘cultural mediator’ ensuring efficient communication between the wet laboratory, the data managers, and the data modelers, should, hopefully in the near future, give way to user (experimentalist) friendly tools, for the generation of intrinsically FAIR data.

## Declaration of Competing Interest

The authors declare that they have no known competing financial interests or personal relationships that could have appeared to influence the work reported in this paper.

## Footnotes

1 FAIRification is the process of making data FAIR.

2 The term metadata is usually defined as data about data. In practice metadata are the piece of data and information that allows a user to understand the aspects of the generation, meaning, and quality of the data.
